# Cross-subtype Immunity against Avian Influenza in Persons Recently Vaccinated for Influenza

**DOI:** 10.3201/eid1401.061283

**Published:** 2008-01

**Authors:** Cristiana Gioia, Concetta Castilletti, Massimo Tempestilli, Paola Piacentini, Licia Bordi, Roberta Chiappini, Chiara Agrati, Salvatore Squarcione, Giuseppe Ippolito, Vincenzo Puro, Maria R. Capobianchi, Fabrizio Poccia

**Affiliations:** * National Institute for Infectious Diseases “Lazzaro Spallanzani,” Rome, Italy

**Keywords:** Heterotypic immunity, vaccination, human influenza, avian influenza, research

## Abstract

Avian influenza virus (H5N1) can be transmitted to humans, resulting in a severe or fatal disease. The aim of this study was to evaluate the immune cross-reactivity between human and avian influenza (H5N1) strains in healthy donors vaccinated for seasonal influenza A (H1N1)/(H3N2). A small frequency of CD4 T cells specific for subtype H5N1 was detected in several persons at baseline, and seasonal vaccine administration enhanced the frequency of such reactive CD4 T cells. We also observed that seasonal vaccination is able to raise neutralizing immunity against influenza (H5N1) in a large number of donors. No correlation between influenza-specific CD4 T cells and humoral responses was observed. N1 may possibly be a target for both cellular and humoral cross-type immunity, but additional experiments are needed to clarify this point. These findings highlight the possibility of boosting cross-type cellular and humoral immunity against highly pathogenic avian influenza A virus subtype H5N1 by seasonal influenza vaccination.

## Abstract 2

Seasonal influenza vaccination may induce heterosubtypic immunity against avian influenza virus (H5N1).

Influenza viruses are segmented, negative-sense RNA viruses belonging to the family *Orthomyxoviridae*. According to the antigenic differences in nucleoprotein and matrix proteins, 3 types of influenza viruses (A, B, and C) have been described. Influenza viruses A and B are associated with seasonal illness and death, whereas influenza virus C causes mild infections (*1*,*2*). Influenza A viruses are subtyped on the basis of the antigenic differences on external hemagglutinin (HA) and neuraminidase (NA) glycoproteins. Human type A influenza virus subtypes have been limited to H1, H2, and H3 and to N1 and N2 (*3*). Several HAs and NAs have been isolated from avian hosts; occasionally, they have been associated with human outbreaks (*4*,*5*).

Cytotoxic T lymphocytes play a central role in the clearance of primary influenza virus infection, peaking after 7–10 days; the peak in antibody titers occurs 4–7 weeks after primary infection (*6*–*8*). Neutralizing antibodies are completely protective against secondary challenges only with closely related strains, but they are ineffective against viruses with major antigenic divergence. For this reason, current influenza vaccines are prepared annually on the basis of World Health Organization forecasts on the most probable influenza A and B virus strains thought to be circulating in the next seasonal outbreak (*5*,*7*). By contrast, cellular responses to cross-reactive epitopes may provide a substantial degree of protection against serologically distinct viruses (*9*). The ability of influenza viruses to mutate and reassort their HA-NA genome segments between different animal species is a main concern because immunity generated by previous infections or vaccinations is unable to prevent infection by itself, although it may reduce virus replication and spread (*8*–*10*).

To date, 3 influenza subtypes have produced pandemic disease in humans: H1N1 in 1918, H2N2 in 1957, and H3N2 in 1968 (*4*,*11*,*12*). In 1997, during the avian influenza (H5N1) outbreak in Hong Kong Special Administrative Region, People’s Republic of China, a cross-reactive cellular immune response induced by influenza (H9N2) was able to protect chickens from influenza (H5N1) (*13*). Moreover, adults living in the United States who were never exposed to H5N1 subtype have shown cross-type cellular immunity to influenza A virus strains derived from swine and avian species (including the H5N1 subtype isolated in Hong Kong) (*14*). Thus, speculation that cross-reactive T cells may decrease illness and death by reducing the replication of the new influenza virus, even if elicited by a different strain, is reasonable.

Avian influenza A viruses of the H5N1 subtype are currently causing widespread infections in bird populations. Numerous instances of transmission to humans have been recently reported in Asia and Africa, with the infection resulting in severe disease or death (>50% fatality rate). Hence, the aim of the present study was to evaluate the immune cross-reactivity between human and avian influenza (H5N1) strains in healthy donors recently vaccinated for seasonal influenza A (H1N1/H3N2). Our data indicate that influenza vaccination may boost cross-subtype immunity against influenza (H5N1), involving cellular or humoral responses or both.

## Study Design and Methods

### Study Population

Healthcare workers wishing to receive seasonal influenza vaccination at the Spallanzani Institute (n = 42) were enrolled. The study was approved by the local Ethical Committee; all participants gave written informed consent. Baseline characteristics of the study population are reported in the Table. Blood samples were obtained before (t0) and 30 days after vaccination (t1). The vaccine formulation was Fluarix, an inactivated and purified split influenza vaccine (GlaxoSmithKline, Verona, Italy). The antigen composition and strains were A/California/7/2004-H3N2; A/New Caledonia/20/99-H1N1; and B/Shanghai/361/2002. Each 0.5-mL vaccine dose contains 15 μg HA of each strain in phosphate-buffered saline and excipients. Vaccine was administered intramuscularly.

**Table:** Baseline characteristics of the study population of healthcare workers, Rome, Italy, 2005

Participant no.	Previous flu vaccine receipt	Sex	Age, y	Work position
1	None	M	37	Biologist
2	None	M	44	Administrative personnel
3	None	M	42	Administrative personnel
4	None	F	27	Laboratory technician
5	2000–2004	M	45	Physician
6	None	F	28	Laboratory technician
7	2002–2004	F	45	Nurse
8	2002–2004	M	59	Laboratory technician
9	2003–2004	M	52	Laboratory technician
10	None	M	48	Nurse
11	None	M	32	Biologist
12	2000–2004	F	42	Physician
13	2000–2004	F	54	Biologist
14	None	M	49	Physician
15	2000–2004	M	55	Physician
16	2001–2004	F	42	Nurse
17	None	F	40	Biologist
18	None	M	38	Biologist
19	None	F	34	Physician
20	2004	M	34	Biologist
21	None	F	28	Biologist
22	2003–2004	M	59	Physician
23	2003–2004	F	42	Physician
24	None	M	51	Laboratory technician
25	None	M	52	Biologist
26	None	M	28	Laboratory technician
27	2003–2004	F	40	Nurse
28	2003–2004	F	32	Nurse
29	2003–2004	M	35	Biologist
30	None	F	51	Laboratory technician
31	2003–2004	M	56	Physician
32	2003–2004	M	36	Physician
33	None	F	32	Nurse
34	2004	M	55	Physician
35	None	F	52	Administrative personnel
36	2004	M	52	Physician
37	2004	M	49	Nurse
38	2004	F	33	Biologist
39	2002–2004	F	44	Nurse
40	2004	F	43	Nurse
41	2004	M	41	Nurse
42	None	M	35	Physician

### Cells, Viruses, and Antigens

Madin-Darby-canine kidney (MDCK) cells were maintained in Dulbecco modified Eagle medium (DMEM) containing 10% fetal calf serum (FCS), and 2 mmol/L L-glutamine, at 37°C in a 5% CO_2_ humidified atmosphere. The influenza (H5N1) virus used was strain A/Hong Kong/156/97 (kindly provided by Paul Chan) (*15*). The virus stock used as challenge antigen in the hemagglutination inhibition (HI) assay was propagated in the allantoic cavities of 10-day-old embryonated hen eggs. The allantoic fluid was harvested 48 h postinoculation and clarified by centrifugation. Virus concentration was determined by HA titration as previously described (*16*), and the virus was stored at –80°C until used. The virus stock used in the microneutralization (NT) and in the cell-mediated immunity assays was propagated in MDCK cells, and the culture supernatants were collected 48 h postinoculation. The 50% tissue culture infectious dose (TCID_50_), determined by titration in MDCK cells, was calculated by the Reed and Muench method (*17*).

Influenza vaccine, UV-inactivated MDCK-derived influenza (H5N1) virus, or synthetic influenza (H5N1) oligopeptides were used as antigens for cell-mediated immunity. Influenza virus (H5N1) was inactivated by exposure to UV light for 10 min, and complete inactivation of UV-exposed virus was checked by infecting MDCK monolayers with undiluted preparation and by back-titrating the infectivity after 5 days postinfection. Four synthetic peptides of the influenza (H5N1) were purchased from Biodesign International (Kennebunk, ME, USA). The sequence of these peptides is specific for H5-C-terminal (15 aa), H5-middle region (14 aa), and N1-C-terminal (15 aa) and for N1-middle region (16 aa), with no cross-matching with other HA and NA sequences. These peptides can bind different HLA-DRB1 alleles, as established according to the SYFPEITHI site (www.syfpeithi.de). Specifically, N1-specific peptides can bind the following HLA-DR alleles: HLA-DRB1*0101, B1*0301, B1*0401, B1*0701, B1*1101, B1*1501. The H5-specific peptide from the N-terminal region binds several HLA-DR alleles (HLA-DRB1*0101, B1*0301, B1*0401, B1*0701, B1*1101, B1*1501). In contrast, the H5-specific peptide from the middle region did not appear to bind any HLA-DR alleles. According to the HLA-DRB1 allele frequency in the local population, these peptides can be efficiently presented by most (up to 84%) of study participants.

### Cell-mediated Immunity

Cell-mediated response was assessed by detecting intracellular interferon-gamma (IFN-γ) production by effector T cells, after antigen-specific stimulation in vitro to generate effector cells from memory cells (*18*). Peripheral blood mononuclear cells (PBMC) were isolated by density gradient centrifugation (Ficoll-Hypaque, Pharmacia Biotech, Uppsala, Sweden) and frozen at –150°C. Thawed PBMC in culture medium (RPMI 1640, 10% FCS, 2 mmol/L L-glutamine) were stimulated with the influenza vaccine preparation (1.5 μg/mL), UV-inactivated influenza (H5N1) (MOI 0.1), or synthetic influenza (H5N1) peptides (NA and HA) (1 μg peptide/mL) for 3 days and expanded for 6 additional days in the presence of recombinant interleukin-2 (IL-2) (5 IU/mL, Boehringer-Mannheim, Mannheim, Germany). On day 9, cells were restimulated with the same antigens in the presence of 1 μg/mL αCD28 and αCD49d (immunoglobulin G1 [IgG_1_], K clones CD28.2 and 9f10, respectively; Becton Dickinson, Mountain View, CA, USA) and of Brefeldin-A (10 μg/mL, Sigma, St. Louis, MO, USA). As a negative control, a mock virus preparation, obtained with uninfected MDCK cells, or irrelevant peptides were used. To control the spontaneous cytokine production, cells incubated with only αCD28 and αCD49d were included.

In addition, the frequency of IFN-γ–producing CD4 T lymphocytes from each donor in the absence of any stimulation was used to calculate the background for each stimulation. The resulting background levels were very low in every experiment, and no differences were observed between samples obtained before (t0 0.03% ± 0.04%) and after vaccination (t1 0.01% ± 0.03%). The frequency of antigen-specific CD4 T cells for each study participant was calculated by subtracting the relative background levels at t0 and t1.

Cell-mediated immunity was considered positive when the net increase was >0.2%. Although retesting samples on separate occasions gave reproducible results, t0 and t1 samples for each participant were tested simultaneously to further reduce test variability.

### Immunofluorescent Staining and Flow Cytometry Analysis

Monoclonal antibodies coupled with phycoerythrin (PE), peridinin-chlorophyll protein (PerCP), allophycocyanin (APC), and phycoerythrin-Cy-7 (PE-Cy7) were combined for simultaneous staining. In this study the following were used: anti-CD4 PerCP (IgG1, clone SK3), anti-CD3 PE-cy7 (IgG2a, clone SK7), anti-IFN-γ APC (IgG1, clone B27), and anti-IL-2 PE (IgG1, clone 5344.111) (Becton Dickinson). Cells were stained as previously described (*19*).

Multiparametric flow cytometry was performed by using a FACSCanto flow cytometer (Becton Dickinson). A total of 300,000 live events were acquired, gated on small viable lymphocytes, and analyzed with FACSDiva software (Becton Dickinson). The instrument was routinely calibrated according to the manufacturer’s instructions.

### Microneutralization and HI Assay

The NT was performed according to a previously described procedure (*20*), in agreement with indications from the World Health Organization (*21*) and the US Department of Health and Human Services (*22*). Specifically, 2-fold serial dilutions of heat-inactivated (30 min at 56°C) human sera were performed in 50 μL DMEM without FCS in 96-well microplates. An equal volume of influenza virus (H5N1) (10^3^ TCID_50_/mL) was then added to each well. Uninfected-cell wells, incubated with each test serum, were included in each plate as negative controls. After 1 h incubation at 37°C, the mixtures were transferred on MDCK cell monolayers and adsorbed at 37°C for 1 h. After washing, DMEM was added, and the plates were incubated for 2 days at 37°C in 5% CO_2_. NT titer was assessed as the highest serum dilution in which no cytopathic effect was observed by light microscope inspection. All serum specimens were tested in duplicate, and t0, and t1 samples from each patient were assayed in the same plate at the same time. The results were scored by persons blinded to the study participant’s identification. The test results were reproducible because random replication of the assays on independent occasions gave consistent results.

The antibody titer was also established by HI test, using for challenge either the seasonal vaccine or the egg-derived influenza (H5N1) preparation. HI assays were performed in V-bottom 96-well plates with 0.5% chicken erythrocytes, as described (*16*).

### Biosafety Laboratory Facilities

All experiments with live highly pathogenic avian influenza A virus (H5N1) were conducted by using Biosafety Level 3-plus (BSL3+) containment procedures (*23*). All investigators were required to wear appropriate masks with HEPA filters.

## Results

### Cell-mediated Immunity to Influenza Viruses

The frequency of circulating antigen-specific CD4 T cells in healthy donors enrolled in the study was analyzed by flow cytometry, by using intracellular cytokine staining assay after the in vitro expansion of effector cells. To generate effector cells from their memory precursors, PBMC were challenged with antigen in vitro for 3 days and expanded for 6 additional days in the presence of IL-2 (*18*).

**Figure 1:**
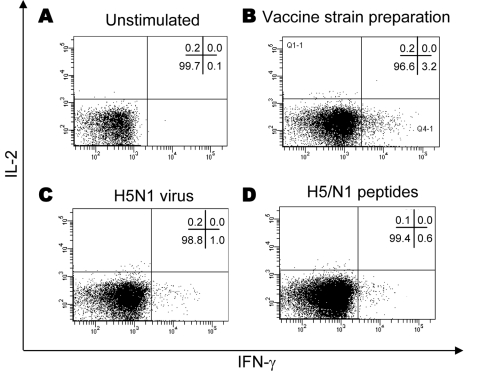
Detection of antigen-specific CD4 T cells against influenza viruses by flow cytometry after in vitro expansion of effector cells. Peripheral blood mononuclear cells were expanded in vitro with interleukin-2 (IL-2) for 9 days in the presence or absence of specific influenza antigens, as indicated, then analyzed by flow cytometry by using the intracellular staining assay. The effector T-cell response was analyzed for interferon-gamma (IFN-γ) or IL-2 cytokine expression. Unstimulated cultures (A), CD4 T-cell response against human influenza vaccine strain preparation (B), inactivated avian influenza (H5N1) (C), and H5/N1 peptides (D) are shown in a representative donor.

Effector cells were characterized for their ability to release IFN-γ when cultured overnight in the presence of antigen. CD4 T cells were gated and analyzed for IFN-γ and IL-2 cytokine expression. A representative experiment with PBMC from a recently vaccinated healthy donor is shown in Figure 1. Without stimuli, no cytokine production in CD4 T cells was detected (Figure 1, panel **A**). However, the stimulation with the seasonal influenza vaccine preparation induced the production of IFN-γ by CD4 effector T cells (Figure 1, panel **B**: 3.2% of IFN-γ+ CD4+ T cells). Stimulation with inactivated influenza (H5N1) virus induced a CD4 T-cell response (Figure 1, panel **C**: 1.0% of IFN-γ+ CD4+ T cells). Finally, some CD4 T cells specific for a pool of H5 and N1 (H5/N1) peptides were also generated in this donor (Figure 1, panel **D**: 0.6% of IFN-γ+ CD4+ T cells). No IL-2 production was observed in these experimental conditions.

### Increased Cell-mediated Immunity after Seasonal Influenza Vaccination

When the extent of CD4 T-cell–mediated immunity before and after seasonal influenza vaccination was compared in the healthy donors enrolled in the study, a nonhomogeneous pattern of responses was detected (Appendix Figure). After vaccination (t1), a 2-fold variation of the frequency of antigen-specific T cells higher than baseline was arbitrarily considered significant. According to this threshold, an increased frequency of IFN-γ–producing CD4 T cells specific for vaccine preparation was observed after vaccination in 5 (donors 8, 11, 17, 26, 42) of 21 donors (23.8%). A slight increase of frequency of the vaccine preparation-specific CD4 T cells was observed in 5 donors (donors 9, 12, 33, 36, 40; 23.8%); a mild-to-significant decrease was observed in the remaining donors (n = 11; 52.3%).

As shown in the Appendix Figure, panel **A**, most donors had a detectable level of vaccine preparation–specific CD4 T cells before vaccination. Six donors (11, 17, 22, 23, 31, 42) had a noteworthy increase over baseline of IFN-γ–producing CD4 T cells specific for the H5N1 subtype (Appendix Figure, panel **B**); among them, 3 were also showing an increase of the frequency of IFN-γ–producing CD4 T cells specific for vaccine preparation (donors 11, 17, 42). Two of them, donor 11 and donor 42, had a significant increase of IFN-γ–producing CD4 T cells specific for H5/N1 peptides (Appendix Figure, panel **C**), which suggests that cross-type immunity may directly involve the HA/NA proteins. Furthermore, 3 other donors (donors 12, 16, 36) had an increased frequency of H5/N1 peptides–specific CD4 T cells, even if they were unable to respond to whole virus.

Indeed, in some persons we also observed a significant decrease at t1 in CD4 T cells specific for vaccine preparation (donors 2, 16, 23, 27, 30, 31, 35, 41), specific for influenza (H5N1) (donors 4, 16, 27), and specific for H5/N1 peptides (donors 2, 27, 34, 39, 41). Donors with a reduced specific response to vaccine preparation at t1 showed a higher frequency of specific CD4 T cells at t0 when compared to other donors (3.4% ± 0.88 vs. 1.29% ± 0.35, respectively, p = 0.013). Similar results were obtained when we observed the influenza virus (H5N1) (1.07% ± 0.47 vs. 0.14% ± 0.03, respectively; p = 0.0093) and H5/N1 peptides (1.19% ± 0.54 vs. 0.13% ± 0.07, respectively; p = 0.0018).

### H5 versus N1 Specificity of the Cell-mediated Response

**Figure 2:**
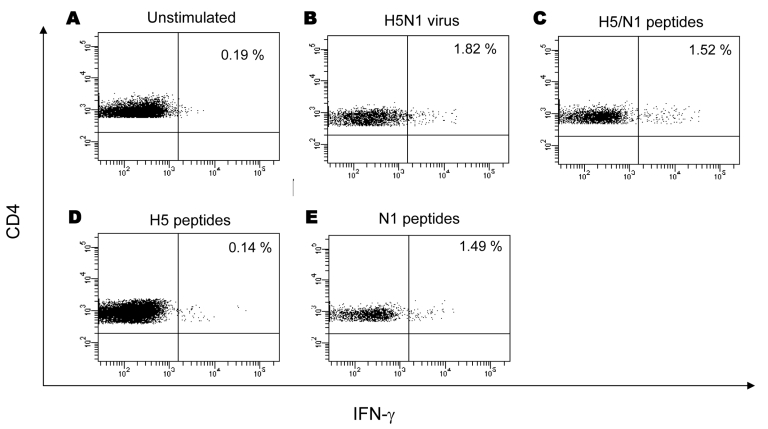
H5 versus N1 specificity of the cell-mediated response. Profiling of influenza (H5N1)–specific CD4 T-cell response in a representative study participant is shown. Peripheral blood mononuclear cells (PBMC) were expanded in vitro in the presence of interleukin-2 (IL-2) and stimulated with inactivated influenza (H5N1) virus (B), peptide pool composed by 4 peptides from H5 and N1 (C), H5 peptides (D) and N1 peptides (E). Panel A shows unstimulated cultures. Dot plots showed the presence, at similar frequency, of specific CD4 T cells when PBMC were stimulated with inactivated influenza (H5N1) virus (panel B, 1.82%), influenza (H5N1) peptides (panel C, 1.52%), and N1 peptides (panel E, 1.49%). No specific CD4 T cells producing interferon-gamma (IFN-γ) were observed after challenge with H5 peptides (D). As negative control, either mock-infected culture supernatants or irrelevant peptides were used, giving results very similar to unstimulated cultures (not shown). A similar pattern was observed in 4 other study participants, supporting the hypothesis that the actual target of cross-subtype immunity could be N1.

Because some study participants were reactive to inactivated influenza virus (H5N1) as well as to a peptide pool composed of 2 peptides from H5 and 2 from N1 consensus sequences, we analyzed whether this reactivity was preferentially directed against HA or NA. As shown by PBMC from a representative donor in Figure 2, the frequency of IFN-γ–producing CD4 effector T cells was appreciable after challenge with the inactivated influenza virus (H5N1) (Figure 2, panel **B**: 1.82% of IFN-γ+ CD4+ T cells) or with the H5/N1 peptides (Figure 2, panel **C**: 1.52% of IFN-γ+ CD4+ T cells). The response of PBMC from the same donor to N1 peptides was positive, whereas the response to H5 peptides was at background level (Figure 2, panel **E** and **D**: 1.49% vs. 0.14% of IFN-γ+ CD4+ T cells), a finding that suggests that N1 seems to be the main target for cell-mediated cross-type immunity against influenza (H5N1) and influenza (H3N2)/(H1N1) vaccine strains. A similar pattern was observed in 4 other study participants, which supports the hypothesis that the target of cross-subtype immunity could actually be N1.

### Increased Humoral Immunity after Seasonal Influenza Vaccination

**Figure 3:**
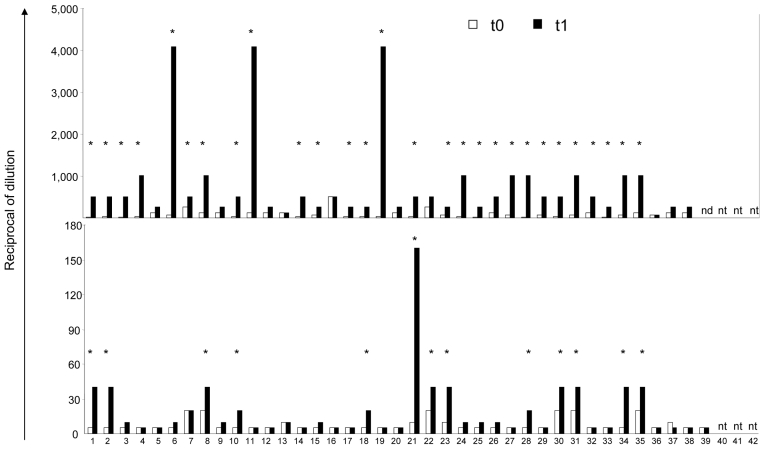
Humoral response against vaccine preparation and influenza virus (H5N1) before (t0) and after (t1) seasonal influenza vaccination. Hemagglutination inhibition (HI) test was used to calculate the antibody (Ab) titer against vaccine preparation (top panel), whereas a neutralization test was used to calculate the antibody titer against influenza (H5N1) (bottom panel) in healthy donors enrolled in the study at baseline (t0) and 1 month after seasonal influenza vaccination (t1). At baseline (white bars), all donors had a detectable level of human influenza antibodies. At t1 (black bars), 28 donors (73.6%) (indicated by *) showed a >4 fold increase of Ab titer against vaccine preparation (HI) over t0. After seasonal influenza vaccination, 13 serum samples (33.3%) (indicated by *) from the study population showed a 20-fold increase of neutralizing Abs against influenza (H5N1) over t0..

Human sera from the same donors were tested for HI activity against both vaccine and influenza (H5N1) preparations and for neutralization activity against influenza virus (H5N1). Individual titers are reported in Figure 3. A 4-fold rise in HA antibody titer is considered noteworthy, and after vaccination most donors (28/38; 73.7%) showed a noteworthy rise of HI titers against vaccine preparation, as indicated by an asterisk (Figure 3, top panel, black bars). HI titers against influenza (H5N1) remained at undetectable levels after seasonal vaccination (data not shown), but a rise of neutralization titer >20-fold over baseline was observed in 13 (34.2%) of 38 donors (Figure 3, bottom panel, asterisk). All but 1 study participant also responded to seasonal vaccination by a rise in HI titers against vaccine preparation. One donor (21) showed high titers against the H5N1 subtype in NT but a low HI titer against vaccine, a unique situation in the study population. However, antibodies to both anti–influenza (H5N1) and influenza vaccine are raised by vaccination. Our findings indicate that seasonal vaccination can raise neutralizing immunity against influenza (H5N1) virus, which shows the existence of an antibody-dependent cross-type immunity. No correlation between influenza-specific CD4 T cells and humoral responses was observed, which suggests that this type of antibody response was mainly CD4 T-cell independent.

## Discussion

We observed that influenza-specific CD4-effector T cells could be generated by long-term cultures in vitro and easily monitored by flow cytometry as IFN-γ–producing cells. When this approach was used, a small frequency of CD4 T cells specific for H5N1 subtype could be detected in several persons at baseline. Seasonal vaccine administration may enhance the frequency of reactive CD4 T cells, boosting the cross-subtype cellular immunity against avian influenza (H5N1). We also observed that seasonal vaccination raised neutralizing immunity against H5N1 subtype in a large number of donors, showing the existence of an antibody-dependent cross-type immunity. Thus, cross-reactive immunity may involve cellular and/or humoral responses, but the humoral response seems to be CD4 independent.

From the present data, N1 appears to be 1 target for cross-type cellular immunity, although we could not rule out the involvement of different (i.e., internal) antigens as possible targets of immune recognition by effector CD4 T cells. Nevertheless, in animal models, cellular immunity (mainly CLT) targeting internal proteins (i.e., NP), partly responsible for heterosubtypic protection, was not induced efficiently by inactivated vaccines (*24*). We did not use live virus, only inactivated split vaccine, whole inactivated virus, or HA and NA peptides for the influenza (H5N1) A/Hong-Kong/156/97 strain. From our data, discriminating between the CD4 T-cell response against external or internal antigens in the case of vaccine preparation was not possible. For H5N1 subtype response, we can presume that the response is against the external antigens and that the results against peptides point to a specific response against NA.

Results obtained with the whole virus and those obtained with the H5 and N1 peptides are not in complete agreement (Appendix Figure). This finding can be explained on the basis of the substantial differences in the antigen presentation underlying the whole virus and peptides. Moreover, we observed that a high activation of specific cells at baseline (t0) was associated with a reduced specific response after vaccination (t1), which suggests that stimulation of pre-activated T cells with high dose of antigen could induce T-cell anergy (*25*) with consequent loss of immune response.

Preliminary evidence also suggests that humoral cross-type immunity is targeting antigens differently from HA: sera from persons showing significant neutralizing titers against influenza (H5N1) did not recognize insect cells expressing HA from the H5N1 subtype (not shown) and did not show HI activity against H5N1 subtype. N1 may possibly also be a target of humoral immunity, but additional experiments such as Western blot analysis or inhibition of NA activity (*26*) are needed to clarify this point.

In animals, exposure to 1 specific subtype of influenza A virus can also induce protective immunity against challenges with other subtypes. This heterosubtypic or cross-protective immunity could represent a key mechanism for facing, and limiting, new influenza outbreaks. In 1997, during the Hong Kong influenza (H5N1) outbreak, an immune response induced by an influenza virus (H9N2), being T cells but not antibodies, protected chickens from lethal influenza (H5N1) (*13*). Moreover, adults living in an urban area of the United States have been described as having influenza-specific memory T cells that recognize epitopes of influenza A virus strains derived from swine and avian species, including the influenza (H5N1) strain involved in the Hong Kong outbreak in humans (*14*).

Our data confirm that persons who have never been exposed to H5N1 subtype may be able to generate a cell-mediated response against the Hong Kong influenza (H5N1) isolate. This cross-type response may be naturally occurring (probably as a consequence of exposure to seasonal influenza strains).

In mice, both CD4 T-cell–independent and –dependent antibody responses contribute to the control of influenza virus infection (*27*,*28*). Although antibodies appear to facilitate the recovery from influenza infection, it is generally believed that B cells cannot produce neutralizing, isotype-switched, influenza-specific antibodies in the absence of CD4 T-cell help (*29*,*30*). However, other data clearly demonstrate that B cells can also produce anti-influenza IgA, IgM, and IgG responses independent of CD4 helper T cells (*27*,*31*). A non–antigen-specific bystander response driven by activated CD4 T cells specific for heterologous antigen may contribute to so-called heterosubtypic immunity (*8*–*10*,*12*). However, the ability of influenza virus infection to promote B-cell activation and differentiation into short-lived, isotype-switched, antibody-secreting cells may result from a combination of B-cell receptor hypercross-linking, engagement of toll-like receptors, production of cytokines, as well as triggering of innate immunity.

In our study, cellular and humoral cross-reactive immunity seemed to target antigens other than HA. Influenza (H5N1) cases occur mainly in young people (*32*). This finding may be explained by hypothesizing that older people, although not previously exposed to H5N1 subtype, may have gained protective immunity by previous infections sustained by circulating influenza virus strains. It has also been shown that immunity to the N1 NA from the human influenza virus cross-reacts with the avian N1 NA virus and that this cross-reactivity protects mice against infection with the avian influenza virus (H5N1) (*26*). All these findings may be explained by hypothesizing that cross-reactive immunity is targeting the N1 NA antigen. However, whether cross-reactive antibodies to NA and CD4 T cells would be protective against illness and death, especially from influenza (H5N1) infection is not known. Further studies will be necessary to elucidate this point.

In conclusion, we demonstrated that vaccination against seasonal influenza may boost a cross-reactive immunity against an unrelated strain responsible for deadly infections in humans, i.e., the avian influenza (H5N1) strain A/Hong Kong/156/97. These data, together with previous experimental results from mice studies and epidemiologic reports, indicate that cross-type immunity should be considered an important component of the immune response against novel influenza A infections.

## Supplementary Material

Appendix Figure Antigen-specific CD4 T cells against vaccine preparation and influenza virus (H5N1) before (t0) and after (t1) seasonal influenza vaccination. The frequency of CD4 T cells producing interferon-gamma (IFN-?) after stimulation with seasonal vaccine preparation (A), inactivated avian influenza (H5N1) (B), and H5/N1 peptides (C) were analyzed in healthy donors enrolled in the study at baseline (t0) and 1 month after seasonal influenza vaccination (t1). At baseline (white bars), all donors had a detectable level of human influenza–specific CD4 T cells; influenza (H5N1)–specific CD4 response was detectable in 41.6% of study participants. At t1 (black bars), some donors (indicated by *) showed an increase of the frequency of antigen-specific CD4 T cells that were twice as high as those in the pre-vaccination level. This increase was arbitrary but considered significant.
