# Supplementary figures and images for: Cross-subtype Immunity against Avian Influenza in Persons Recently Vaccinated for Influenza
Source: Emerg Infect Dis. 2008 Jan;14(1):121–8. doi: 10.3201/eid1401.061283 (PMC2600140; doi:10.3201/eid1401.061283)

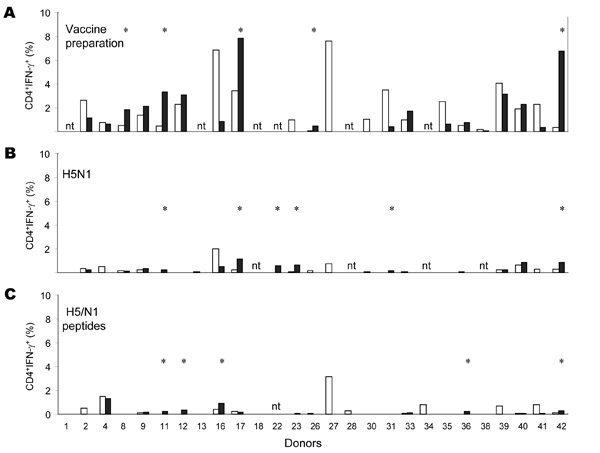

Supplement: Appendix Figure — Antigen-specific CD4 T cells against vaccine preparation and influenza virus (H5N1) before (t0) and after (t1) seasonal influenza vaccination. The frequency of CD4 T cells producing interferon-gamma (IFN-?) after stimulation with seasonal vaccine preparation (A), inactivated avian influenza (H5N1) (B), and H5/N1 peptides (C) were analyzed in healthy donors enrolled in the study at baseline (t0) and 1 month after seasonal influenza vaccination (t1). At baseline (white bars), all donors had a detectable level of human influenza–specific CD4 T cells; influenza (H5N1)–specific CD4 response was detectable in 41.6% of study participants. At t1 (black bars), some donors (indicated by *) showed an increase of the frequency of antigen-specific CD4 T cells that were twice as high as those in the pre-vaccination level. This increase was arbitrary but considered significant. [file 06-1283_app-s1.gif]
